# Condensations from Late Editorials in the Medical Press

**Published:** 1881-07

**Authors:** 


### Condensations from Late Editorials in the Medical Press.

---

NO PREMIUM ON FOLLY.—Not long ago an American physician lost his life from diphtheria contracted in the attempt to clear a tracheotomy tube by sucking it. His act of folly was, in the opinion of some, hardly condoned by its punishment; by others it was lauded as an act of heroism. A recent number of a contemporary relates with satisfaction, that the Albert medal has been conferred upon a young surgeon of England—a Mr. Grie'—who applied his mouth to the tube to restore respiration in a similar case. The editor of our contemporary makes this the occasion for some ironical remarks about the want of appreciation of such deeds in "the land of the free and the home of the brave."

It is a pity, not that there should be this lack of appreciation, but that there should be any exception to it. It is time, indeed, that expressions of admiration for reckless and unthinking acts of folly should stop, and that men who have any influence in molding public sentiment should strengthen the opinion that it is not praiseworthy, but on the contrary censurable, for one to expose a valuable life in the hope of saving one of less value, and tenfold reprehensible when the risk is so enormous and the benefits are so unlikely to be obtained or kept as in the cases we have just referred to. The man who does such things is not brave, he is rash; and, hard as it may sound, the American who lost his life and the Englishman who received the Albert medal were both actuated by idiotic although courageous impulsiveness.—*Medical Times*, April 23.

CONVALESCENT HOSPITAL.—A daily contemporary recently called attention to the case of a poor consumptive, who shot himself after failing to obtain admission to a hospital because of the incurability of his disease. The paper in question takes occasion to refer to the disposition on the part of hospitals to refuse admission to incurables, and casts unpleasant reflections upon the motives which actuate them in so doing. There is no denying the fact that the majority of our hospitals are adverse to admitting incurables, simply for the reason that thereby help would be denied to many other cases which are curable, and which might otherwise die.

What is needed is a convalescent hospital arranged on an economical plan, furnished with the ordinary conveniences of a home, and supplied with medical attendance. No costly accommodations are required, neither would it be necessary that the buildings be situated in the city, on expensive ground. Hospitals could be built on the cottage plan in suburban districts, and be made to a certain extent self-supporting by the light work that could be easily performed by the majority of the patients. It seems strange, in view of the great good to be accomplished by the establishment of such an institution on a sufficiently comprehensive plan, that some definite means have not been taken to that end.—*Medical Record.*

---

MEDICAL, AS COMPARED WITH LEGAL FEES.—Whose fault is it that doctors are, as a rule, so poorly paid? Chiefly, it is the fault of the doctors themselves. First, in that they charge so little; second, in that they so seldom bring suits for recovery; and third, in that they detract from the value of each other's service. The gentlemen of the law are not guilty of any of these misdemeanors, and the victim of even an exhorbitant fee would as soon think of resisting, defenseless, a whole gang of assailants, as of disputing a bill for legal services.

Doctors, on the other hand, simple, foolish fellows, do not support each other at all when it comes to the matter of fees. They feel insulted when they hear that a fellow-practitioner has received an unusual fee. They never got that much themselves, and they know their own equal, if not superior skill. A favorite expression of a little envy is to raise the cry on a fellow-practitioner of "high charges." The regulars get the "regular" fees, and the big fees go to the quacks.

Another difficulty in the matter of fees comes from the side of the law. The gentlemen of the law have the highest appreciation of the value of their own precious services, but the lowest of that of medical men. Honorable judges on the bench award to the meanest lawyers for the cheapest kind of service round sums from private or public funds, while skilled practitioners of our art are not even paid for their loss of time. The young fledgling attorney will receive more pay from the city funds for a few days' defense of a vile criminal, by order of the court, than a ward physician in a year for taking care of the honest poor.

When Prof. Charcot received $9,000 for a consultation, implying a trip from Paris to Moscow, it was thought worthy of note all over the world. Well, we know of a fee of $40,000 received by an attorney at law in this city, for services in a case unknown outside the city limits. Five thousand dollars were paid to the attorneys, in Covington, for the settlement of an estate of $210,000, all in personal property, equal to cash, and but one-fourth that sum to the two physicians who swore to three years' continuous service. And yet a third physician, called once in consultation in the case, refused to sign a paper certifying to the correctness of the medical bill, unless the two physicians would declare that every service had been jointly made.

May the day never come—and it never will—when medicine will merit the opprobrium which now rests, in the matter of money, upon the law, but may it come soon when a fairer recompense for service, whose effect upon the mind and body no one but a physician knows, will be granted without a grudge.—*The Cincinnati Lancet and Clinic*, April 30.

---

THE ONTARIO MEDICAL ACT.—An American graduate, if we understand the Ontario Act aright, is to make "his education equivalent to that required of graduates in Canadian schools, passing the same matriculation, and attend the same number of six months' winter courses of lectures." Hence the American graduate, at the end of his three years of college study, of four and a half to five months, plus the period and assiduousness of his study at home during the remainder of each of the three years, cannot enter upon the practice of medicine—whatever his attainments—in Canada, unless he attends a fourth year in some Canada school. Such a law as this would require some of the leading medical lights of the world to go to school again. Think of Marion Sims, Robert Battey, Thomas, Barker, Emmet, Goodell, Flint, junior and senior, Loomis, Sayre, Draper, Dalton, Stillé, Bartholow, Hammond, Hamilton, Mitchell, Wood, Gross, etc., sitting as students in the Canadian schools of medicine!

From the correspondence we have had with doctors of the United States, and which we have seen from Canadians, we are free to say we do not believe any *intentional* discourtesy has been done the regular graduates of the recognized medical colleges of the United States by the *regular* practitioners of the Dominion. Yet, while this may be so, in view of all the evidence we have in hand, we still think there are national professional courtesies between Canada, as well as Mexico, and the United States—courtesies of a character which we have tried to indicate—which should always be observed. If any well-recognized Canadian or Mexican practitioner of medicine comes to the United States to see a patient, whether he be called in consultation or to attend a patient, let the courtesy be extended of allowing that recognized practitioner to come. Or, if the summons of similar character be sent a regularly recognized practitioner of the United States, to go to either of the classes of patients in either of these different countries, let him be accorded the courtesies of the medical profession, at large, throughout the civilized world, without arrest, or even the threatening of arrest by the courts, the police, or any member of the profession.

If such indignities as have, on several occasions been offered "regular doctors" of the United States, are persisted in, it will become proper for the Congress of the United States to enact some self-protective law in the premises. We trust the occasion for such a Congressional enactment will never occur.—*Virginia Medical Monthly*, May.

The same medical law is enforced in the Provinces of Quebec and New Brunswick. What is needed is a clause in the Medical Act which would allow any foreign graduate of medicine to register, after passing a satisfactory examination before a board appointed for the purpose by the legislature or the medical colleges of Canada.

---

THE SOCIAL STATUS OF THE MEDICAL PROFESSION.—It has always been a grievance with the profession in Europe, that its social position has been placed too low. Of the three so-called "learned" professions it ranks the lowest, and it never has pretended to equal the military or diplomatic service. The bar and the pulpit, the sword and the gown, have, in all periods of English history, overawed the medical man.

There is reason in this. The medical man is a manual laborer, a "chirurgeon," literally a hand-worker; his proceedings are "maneuvers," works of the hand; his art is that of a skilled laborer whose success lies in his manual dexterity; and his work is none of the cleanest. Another reason is that he is a tradesman. His hand is extended for the shilling which he earns. In this immediate grasping for money, there is believed to be something deteriorating, and so there is.

Not long since, Dr. J. Milner Fothergill excited the intense ire of some of his associates by describing, in a series of letters to an American contemporary, the present low social position of the country doctor in England. In fact his statements were correct.

It is not better in Germany. The physician there is far from holding an equality with even the inferior nobility. It has been a matter of wondering comment that a few have married into the higher classes. Last year Dr. Wilin, an eminent physician of Breslau, married a Princess of Wurtenberg, greatly to the disgust of her relatives, no one of whom, probably, was his equal in education and mental vigor. The celebrated surgeon, Prof. Esmarch, is another of the few instances.

Such examples are extremely rare. Probably it is in France that the distinguished physician obtains the highest social awards. There is a directness about the French mind which seizes the relations of social life more quickly than any other.

In this democratic country of ours such invidious distinctions do not obtain. There are no discriminations against any honest avocation. The man is judged by his merits and the success which they bring him. He gains whatever social life has to offer without difficulty, if he makes it an object to do so. He is little the better for belonging to a learned profession, and certainly nothing the worse. This, we may take it, is the proper light for him to be viewed in.—*Medical and Surgical Reporter*, April 23.

---

SMALL-POX IN LONDON.—The fatal cases of small-pox in London, which had steadily increased from forty-three to seventy-seven in the five preceding weeks, further rose last week to eighty-four, and exceeded the corrected average number in the corresponding weeks of the last ten years by thirty; thirty-nine were recorded in the Metropolitan Asylum Hospitals at Fulham, Homerton, Stockwell, and Deptford, six in the Highgate Small-Pox Hospital, and thirty-four in private dwelling houses. Of the eighty-four persons who died from small-pox within registration London, thirty-two had resided in the South, twenty-four in the East, nineteen in the North, five in the West, and three in the Central groups of registration districts. The eighty-four fatal cases included twenty-three of children under five years of age, twenty-four of persons aged between five and twenty years, twenty-six of persons aged between twenty and forty years, and eleven of persons aged upwards of forty years. The number of small-pox patients in the Metropolitan Asylums Hospitals, which had increased from 820 to 940 in the three previous weeks, further rose to 963 on Saturday last. The new cases of small-pox admitted to these hospitals, which had been 224 and 244 in the two previous weeks, were 227 last week. The Highgate Small-pox Hospital contained ninety-five patients on Saturday last, against seventy-seven and eighty-five at the end of each of the two preceding weeks; twenty-nine new cases were admitted to this hospital during the week.—*British Medical Journal*, April 30.

---

THE PLAIN TRUTH ABOUT VIVISECTION.—The appearance of a new bill bearing on its back the names of Sir Eardley Wilmot, Mr. Samuel Morley, and Mr. Firth, and having for its specific object the *total* abolition of vivisection, supplies us with a not unwelcome opportunity of placing our views explicitly before the profession and the public. In short, an occasion has arisen for speaking the plain truth about vivisection, and we shall not shrink from uttering it.

Vivisection is a scientific necessity; and, inasmuch as it is just as idle to try to stay the progress of science as it is to oppose the rising tide on the sea shore, the attempt to suppress this particular mode of investigation cannot be ultimately successful. Notwithstanding the assertions made by men with warm and feeling hearts, it is an incontrovertible fact that nearly all we *know* of the living organism in health and disease has been learned by vivisection, either intentionally performed, or, as the phrase goes, "accidentally" accomplished. Vivisection has been practiced in all ages and countries, more or less extensively. It is the analytical method applied to the study of organic life, just as the demolition of the earth's crust by the geologist's hammer is the analytical method applied to inorganic nature. The fact that the living animal feels is an untoward contingency, but it cannot qualify the major consideration. The dissection of the dead organism is tributary or complementary, and may well be made preparatory, to the dissection of the living body; but in the long run we must find ourselves face to face with the grim truth that it is the *living* body which science requires, and sternly demands, to explore, and which sooner or later she will explore in spite of all the hindrances which sentiment and humanity may throw in her way. If vivisection is totally abolished in this country practical physiologists will have to go elsewhere. The great bulk of the information which has enabled physicians to treat diseases with new precision in recent years, and which has already led to the lengthening of many useful lives, is the outcome of vivisection. The aim to minimize the recourse to it was humane, the attempt to abolish it altogether is absurd.

The bill before us writes it own epitaph in the seventh clause: "This Act shall not apply to inter-vertebrate animals." If no pain is to be inflicted in the interests of science we must desist from vivisection of every kind. It is a contemptible affectation of pharasaic humanity to take credit for protecting the dog while we allow the frog to be dismembered for our information and advantage. If a more stringent act on this subject were passed to-morrow, the average number of vivisections now performed would not be reduced. The legislative exploit would simply be offering a little additional incense to our national vanity. The operations denounced here would be performed elsewhere; and then, as now, we should hug the comforting sense of superior humanity, while we bid eagerly for the increased information science might gain in her "cruel" quest.—*London Lancet*, May.

---

THE "HAMMOND PRIZE" OF THE AMERICAN NEUROLOGICAL ASSOCIATION.—The American Neurological Association offers a prize of five hundred dollars, to be known as the "William A. Hammond Prize," and to be awarded at the meeting in June, 1882, to the author of the best essay on the *Functions of the Thalamus in Man.*

The conditions under which this prize is to be awarded are as follows:

1. The prize is open to competitors of all nationalities.

2. The essays are to be based upon original observations and experiments on man and the lower animals.

3. The competing essays must be written in the English, French or German language; if in the last, the manuscript is to be in the Italian handwriting.

4. Essays are to be sent (postage prepaid) to the Secretary of the Prize Committee, Dr. E. C. Seguin, 41 West 20th street, New York City, on or before February 1, 1882; each essay to be marked by a distinctive device or motto, and accompanied by a sealed envelope bearing the same device or motto, and containing the author's visiting card.

5. The successful essay will be the property of the Association, which will assume the care of its publication.

6. Any intimation tending to reveal the authorship of any of the essays submitted, whether directly or indirectly conveyed to the Committee or to any member thereof, shall exclude the essay from competition.

7. The award of the prize will be announced by the undersigned Committee; and will be publicly declared by the President of the Association at the meeting in June, 1882.

8. The amount of the prize will be given to the successful competitor in gold coin of the United States, or, if he prefers it, in the shape of a gold medal bearing a suitable device and inscription. Signed:

F. T. MILES, M.D., Baltimore.
J. S. JEWELL, M.D., Chicago.
E. C. SEGUIN, M.D., New York.

---

BY the opening of the fourth course of lectures in October next, the Washington Training School for Nurses hopes to be able to rent a building and have a comfortable house for the nurses, where their training may be systematically conducted under an experienced and educated head nurse, and where the lectures may be given by the medical faculty until a general hospital is established, in which the training of nurses will, we trust, be made a legitimate part of its functions. As an encouragement to those who contemplate entering upon the calling of the "trained nurse," they can state that all of their advanced pupils have found in Washington full and remunerative employment even before they had finished their studies, and only consented to assume the responsibility under the most urgent appeal, but they have in every case given satisfaction alike to the sick and the physician in attendance.—*Dr. Toner.*

